# The Influence of Aging on the Functional Connectivity of the Human Basal Ganglia

**DOI:** 10.3389/fnagi.2021.785666

**Published:** 2022-01-12

**Authors:** Clara Rodriguez-Sabate, Ingrid Morales, Manuel Rodriguez

**Affiliations:** ^1^Center for Networked Biomedical Research in Neurodegenerative Diseases, Madrid, Spain; ^2^Laboratory of Neurobiology and Experimental Neurology, Department of Basic Medical Sciences, Physiology, Faculty of Medicine, University of La Laguna, San Cristóbal de La Laguna, Spain

**Keywords:** aging, basal ganglia, functional connectivity, hand motion, resting state

## Abstract

Although basal ganglia (BG) are involved in the motor disorders of aged people, the effect of aging on the functional interaction of BG is not well-known. This work was aimed at studying the influence of aging on the functional connectivity of the motor circuit of BG (BGmC). Thirty healthy volunteers were studied (young-group 26.4 ± 5.7 years old; aged-group 63.1 ± 5.8 years old) with a procedure planned to prevent the spurious functional connectivity induced by the closed-loop arrangement of the BGmC. BG showed different functional interactions during the inter-task intervals and when subjects did not perform any voluntary task. Aging induced marked changes in the functional connectivity of the BGmC during these inter-task intervals. The finger movements changed the functional connectivity of the BG, these modifications were also different in the aged-group. Taken together, these data show a marked effect of aging on the functional connectivity of the BGmC, and these effects may be at the basis of the motor handicaps of aged people during the execution of motor-tasks and when they are not performing any voluntary motor task.

## Highlights

- The effect of aging on the basal ganglia interactions was studied with neuroimaging methods.
- Aging deteriorates the functional connectivity of basal ganglia.
- Basal ganglia are involved in the motor disorders of aged people.

## Introduction

The decline of motor abilities associated with aging normally occurs parallel to changes in different cortical and subcortical motor centers (Fjell and Walhovd, 2010; Seidler et al., 2010). Although changes in the volume (Seidler et al., 2010; Walhovd et al., 2011) and structural connectivity (Bhagat and Beaulieu, 2004; Wang et al., 2010; De Groot et al., 2015; Cox et al., 2016; Behler et al., 2021) of basal ganglia (**BG**) may be involved in age-associated motor deterioration, the actual role of these changes has not been clearly established. A circumstance that limits the association of the motor handicaps and the BG changes induced by aging is that although motor deterioration is better known (Sun et al., 2012; Ferreira and Busatto, 2013; Mathys et al., 2014; Sala-Llonch et al., 2015; Xiao et al., 2018), the effect of aging on the functional connectivity of BG is less clear. The study of BG activity with magnetic resonance imaging (**MRI**), and particularly with functional connectivity MRI (**fcMRI**), has reported inconsistent results indicating an increase (Marchand et al., 2011), a decrease (Taniwaki et al., 2007) or no changes (Baudrexel et al., 2011) in the functional connectivity of BG with aging. This low consistency of fcMRI studies may be associated with age-related changes in the neuro-vascular coupling (Riecker et al., 2003), the small size of some BG (which hampers the grouping of data obtained in different subjects), and the closed-loop wiring of BG (the interaction between two centers may reflect the circulation of information across the BG closed-loop circuit more than their direct interaction).

This work was planned to study the influence of aging on the functional connectivity of the BG regions directly involved in the execution of movements, the BG nuclei included in the BG motor circuit (**BGmC**). BGmC is a closed-loop circuit composed of projections from the primary motor cortex (**M1**) to the posterior regions of the putamen (**Put**), and from this center to the external globus pallidum (**GPe**), subthalamic nucleus (**STN**), internal globus pallidum (**GPi**), substantia nigra (**SN**), and the motor thalamus (**MTal**), a thalamic region that sends projections back to the M1 and completes the closed-loop circuit of BG (Alexander et al., 1986; Hoover and Strick, 1993; Delong and Wichmann, 2009). In order to prevent the fcMRI computed between two centers from being “contaminated” by their common interactions with other BG, data used to study the interaction between each two BG were “regressed” with data recorded in all the other BG (partial correlation) (Zhang et al., 2008, 2010). In order to prevent the “contamination” of the blood-oxygen-level-dependent (**BOLD**) data of the smallest BG (e.g., STN) by those of surrounding structures, the data used to represent the activity of each center was computed by averaging voxels included in a volume-of-interest (**VOI**) which was located inside each BG of each subject according to previously reported procedures (Rodriguez-Sabate et al., 2015, 2017b). The partial correlation coefficient (**CC**) was used to estimate the “magnitude” of the functional interaction of BG (Fox and Raichle, 2007), and a block-task paradigm with interleaved “no-motion”/“motion” (hand movements) intervals was used to study the influence of motion on the BG interaction (Fair et al., 2007).

## Methods

### Subjects

Thirty healthy volunteers 20–67 years of age (15 men and 15 women; 45.1 ± 12.6 years old; mean ± standard deviation) showing: (1) no acute or chronic illness, (2) no history of neurological diseases (they showed a normal neurological examination and no evidence of motor disorders according to the Hoehn and Yahr, the Schwab and England scales), (3) no history of psychiatric diseases (including no evidence of dementia and normal values in the Montreal Cognitive Assessment and the Mini Mental State examinations), (4) normal values in basic laboratory tests, and (5) Normal MRI scans. Written informed consent was provided by all participants, and all procedures were in accordance with the ethical standards of the Declaration of Helsinki. The study was approved by an institutional review board (Human Studies Committee-La Laguna University). Subjects were divided into two groups, the young-group (26.4 ± 5.7 years old; 8 men with an average age of 25.4 and 7 women with an average age of 27.3) and the aged-group (63.1 ± 5.8 years old; 7 men with an average age of 61.7 and 8 women with an average age of 64.5).

### Data Collection

The basic experimental procedures were similar to those previously reported (Rodriguez-Sabate et al., 2015, 2017a). Briefly, the BOLD-fluctuation of BG was used to study the functional connectivity of the BGmC in subjects who performed a motor-task or remained at rest. A block-task paradigm with interleaved “no-motion”/“motion” intervals was used. During the motor-task block, subjects performed a repetitive sequence of finger extensions/flexions with the right-hand (from the little finger to the thumb and back to the little finger). During the no-motion block, subjects did not perform any planned task. The transitions between the no-motion and motion time intervals were orally announced by a single word, “MOVE” to start motion and “STOP” to finish motion. One hundred volumes were recorded in each of the four task-blocks. In order to prevent the effects of the transitions between tasks (the change of the BOLD signal baseline may need seconds) the frames 1–10 of each block were not included in the data analysis.

BOLD-contrast images (64 × 64 sampling matrix with voxels of 4 × 4 × 4 mm) were acquired (GE; 3.0 T) in a coronal plane (250 × 250 mm field of view) with gradient-echo (echo-planar imaging; repetition-time 1,600 ms; echo-time 21.6 ms; flip-angle 90°). fMRI data were co-registered with 3D anatomical images (repetition-time 7.6 ms; echo-time 1.6 ms; flip-angle 12°; 250 × 250 mm field of view; 256 × 256 sampling matrix; voxels of 1 × 1 × 1 mm). Functional and anatomical studies were obtained in a single session and with the head fixed in the same position. Different structural markers were jointly used to decide where to place each ROI in the brain of each subject (Rodriguez-Sabate et al., 2017b). Briefly, ROIs were positioned in each center of each subject by using the Talairach coordinates, the shape of the nucleus, and the anatomical relationship of the nucleus with other structures (external cues) as the main indicators (working with normalized 3D-anatomical images). All centers were identified in coronal slices located 4–27 mm posterior to the anterior commissure. The optic tract, internal capsule, and medial forebrain bundle were used as external cues to identify the putamen, GPe, GPi and MTal. GPi was initially identified ≈6 mm posterior to anterior commissure and just over the optic tract. The ROI for the putamen was located at post-commissural level because the somato-sensorimotor regions primarily project to the posterior putamen. The post-commissural putamen was then identified ≈5 mm posterior to anterior commissure. GPe was located ≈3 mm posterior to the anterior commissure. MTal was located ≈11 mm posterior to anterior commissure (5 mm posterior to the GPi). Special care was taken to identify the STN. Three external cues were used to identify the STN, the oculomotor nerve, cerebral peduncle, and pons. Coronal images were initially moved backwards and forwards (between 10 and 18 mm posterior to anterior commissure) to identify the slice where the oculomotor nerve was trapped in the most medial region of the contact between the pons and cerebral peduncle. The backward-forward movements of the slices were also used to identify the slice where the oculomotor nerve was trapped between the pons and cerebral peduncle. The STN was identified, in this slice, as the center located 10 mm medial to the optical tract, just above a horizontal line crossing this tract and near the medial boundary of the cerebral peduncle. The STN, in this position, was mainly surrounded by tracts and located anterior to the SN. So as not to mix data on specific centers with those of the surrounding centers, the ROIs were generally small and clearly located within each nucleus. This was not the case of the SN. In humans, SN pars compacta is intermixed with SN pars reticulata and both portions of the SN cannot be clearly segregated in MRI images. Thus, the ROI in this case included the whole SN, and was located between the red nucleus and posterior commissure in slices −22 to −26 mm posterior to the anterior commissure. The M1 representation of the hand was located in the precentral gyrus, just posterior to the junction of the superior frontal sulcus with the precentral sulcus. Depending on the slice level, the hand representation in M1 has a “zig-zag” or “step-like” shape or a “hook” or “Ω” shape. This distribution was generally observed in a position anterior to the protrusion (“knob”) of the precentral gyrus toward the central sulcus. The fMRI response was also used to verify the location of M1 (10 voxels showing the maximum BOLD response to finger movements) because the comparison of the BOLD signal between the no-motion and the motion intervals clearly showed an activation in hand representation in M1. This is a time consuming method, but it prevents the mixture of different brain regions when BOLD-data are integrated in an experimental group. All data sets were normalized to the Talairach space.

### Data Pre-processing

Data were preprocessed (BrainVoyager software) with a slice scan-time correction, a 3D-motion correction, and a temporal filtering (0.009 Hz high-pass GLM-Fourier filter). No spatial smoothing was performed, and studies with a brain-translation >0.5 mm or a brain-rotation >0.5 degrees were rejected. Residual motion artifacts and physiological signals (respiration, cardiac activity) were diminished by regressing the BOLD-signals with the mean average of the BOLD-signals recorded in white matter and brain ventricles (Power et al., 2014).

### Correlation Methods

fcMRI was computed with the mean BOLD-signal of voxels included in each VOI, and values of right and left brain centers were grouped together (Gopinath et al., 2011). The Pearson correlation coefficient (*r*; *p* < 0.001 two-tailed) was used to estimate the “strength” of the functional connectivity of the BG centers (Statistica-Statsoft, Tulsa). Partial correlations were used to eliminate the collateral influence of the other BG (used as “regressors”) on the functional connectivity between two particular centers, a method which is particularly useful in closed-loop networks where the activity of any center may have time-relationships with the activity of all the other centers of the network (Zhang et al., 2008, 2010).

The motor-task effect and the aging effect on the interaction between two centers were considered to be significant when the change of the partial correlation computed for their BOLD-signals reached statistical significance. Differences between two correlation coefficients were identified by using the r-to-Fisher-z transformation (*r*′ = 0.5^*^(ln(1 + *r) – ln(1-r); r*′ *being the Fisher-z transformed r)* and a two-sided *t* comparison (*mean and standard error of each sample evaluated against the t distribution with df* = *n1* + *n2 – 2 degrees of freedom; n1 and n2 being the sample sizes*) adjusted for multiple comparisons (Greicius et al., 2003).

## Results

Table 1 shows the position and size (no. of voxels) of VOIs used to characterize the BOLD activity of BG. No statistical difference was found between the VOI sizes in the young and aged groups.

**Table 1:** Coordinates are shown in mm (Talairach).

	**X lateral**	**Y posterior**	**Z superior**	**Size**
**Primary motor cortex**				
Young-group	31.1 ± 3.8	−19.6 ± 5.2	53.2 ± 6.4	40.7 ± 8.9
Aged-group	37.3 ± 3.9	−19.7 ± 5.3	48.3 ± 5.3	37.2 ± 10.9
**Putamen**				
Young-group	26.7 ± 1.8	−4.8 ± 1.2	0.33 ± 0.1	21.9 ± 4.1
Aged-group	27.1 ± 1.3	−5.2 ± 1.3	0.41 ± 0.4	20.1 ± 3.7
**External pallidum**				
Young-group	14.6 ± 5.5	−2.4 ± 0.6	2.0 ± 1.8	8.5 ± 4.0
Aged-group	16.8 ± 1.9	−2.3 ± 1.3	2.8 ± 2.2	7.4 ± 3.7
**Internal pallidum**				
Young-group	13.7 ± 2.1	−6.4 ± 1.1	−2.1 ± 1.7	8.2 ± 0.6
Aged-group	14.9 ± 1.8	−6.1 ± 1.7	−1.4 ± 1.9	8.2 ± 0.6
**Subthalamic nucleus**				
Young-group	11.2 ± 1.5	−13.8 ± 2.0	−3.3 ± 2.5	11.3 ± 4.6
Aged-group	10.8 ± 1.7	−13.1 ± 2.2	−5.1 ± 2.7	12.1 ± 3.6
**Substantia nigra**				
Young-group	7.3 ± 1.6	−19.2 ± 1.3	−7.9 ± 1.7	224.1 ± 31.1
Aged-group	7.2 ± 0.7	−18.5 ± 1.3	−9.1 ± 3.1	217.1 ± 29.3
**Ventral-anterior thalamus**				
Young-group	9.1 ± 0.7	−11.0 ± 1.0	6.6 ± 3.0	22.6 ± 6.2
Aged-group	9.6 ± 1.2	−11.1 ± 1.6	7.5 ± 1.9	28.2 ± 7.3

*The size of the VOIs is shown by the number of their structural voxels*.

### Functional Connectivity of BG During the No-Motion Intervals

**Figure 1:**
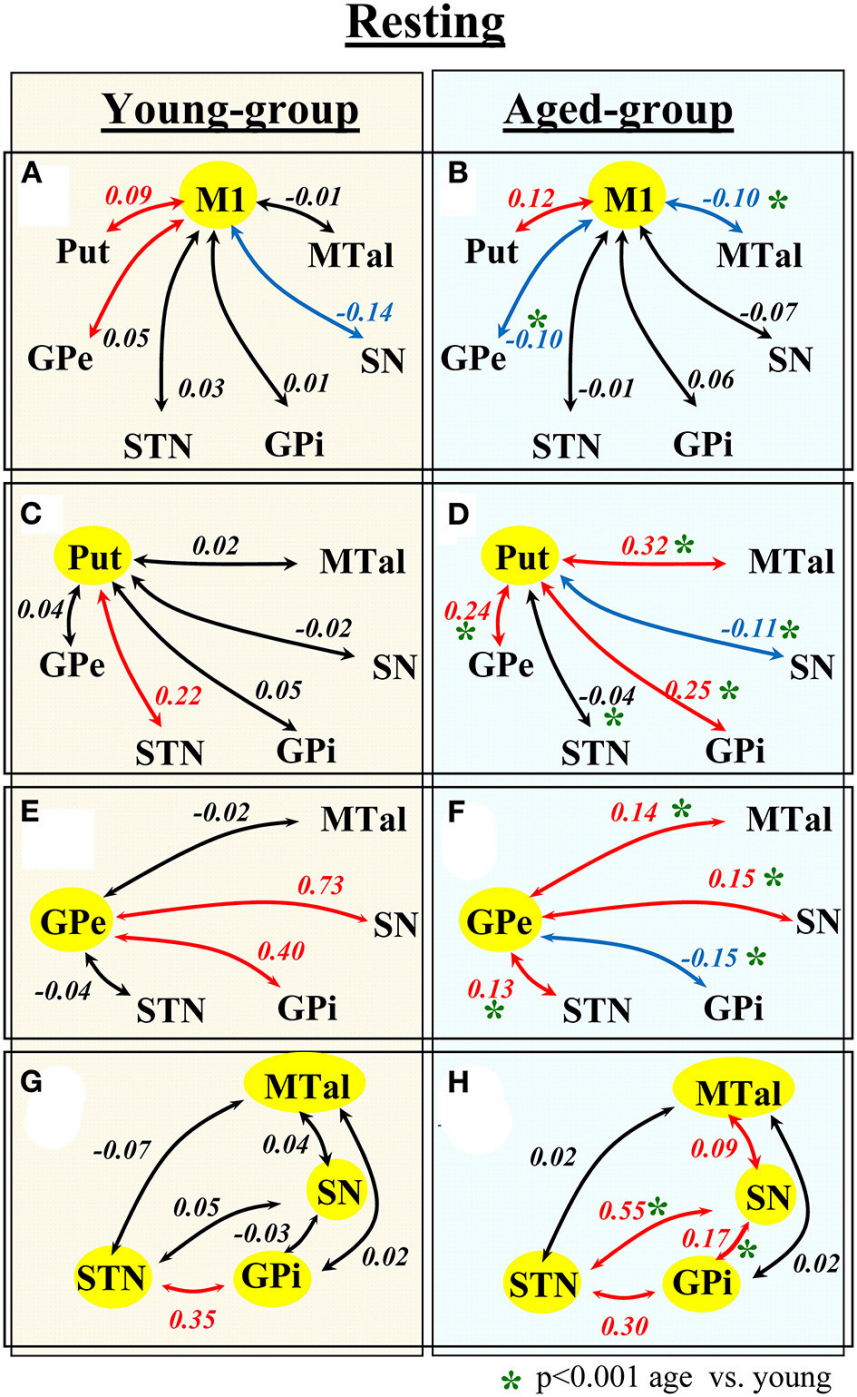
Functional connectivity of BG during task-resting in the young- and aged-groups. Diagrams show the partial correlation coefficient in the young-group (left-hand side) and aged-group (right-hand side). Numbers represent the mean correlation coefficient, in red positive correlations, in blue negative correlations and in black no statistical correlations. Statistical differences between the correlation computed in the young- and aged-groups are shown by green asterisks. M1, primary motor cortex; Put, post-commissural putamen; GPe, external globus pallidum; STN, subthalamic nucleus; GPi, internal globus pallidum; SN, substantia nigral; and MTal, motor thalamus.

Figure 1 shows the functional connectivity during the no-motion intervals (indicated by the partial correlation coefficient CC) in the young (left) and aged (right) groups. In the **young-group**, the M1 BOLD-activity showed a significant positive CC with the Put and GPe, and a negative correlation with the SN (Figure 1A). Positive correlations were also found between Put-STN (Figure 1C), GPe-SN (Figure 1E), GPe-GPi (Figure 1E), and STN-GPi (Figure 1G). The GPi, SN, and MTal did not showed any significant correlation between them (Figure 1G).

The functional connectivity in the **aged-group** was clearly different to that observed in the young-group. In the aged-group, the **M1** showed a significant positive correlation with the Put, and a negative correlation with the GPe and MTal (Figure 1B). The **Put** showed a positive correlation with the GPe, GPi, and MTal and a negative correlation with the SN (Figure 1D). The **GPe** showed a positive correlation with the STN, SN and MTal, and a negative correlation with the GPi (Figure 1F). The **STN** showed a positive correlation with the GPi and SN (Figure 1H). The **SN** showed a positive correlation with the GPi and MTal (Figure 1H). When compared with the young-group, the aged-group showed a significant increase of positive correlations between Put-GPe, Put-GPi, and Put-MTal (Figures 1C,D), GPe-MTal and GPe-STN (Figures 1E,F), STN-SN, SN-GPi and SN-MTal (Figures 1G,H), and a significant increase of negative correlations between M1-MTal, M1-GPe (Figures 1A,B) and Put-SN (Figures 1C,D). Some positive correlations found in the young-group vanished (Put-STN; Figures 1C,D) or were replaced by negative correlations (GPe-GPi in Figures 1E,F) in the aged-group.

**Figure 2:**
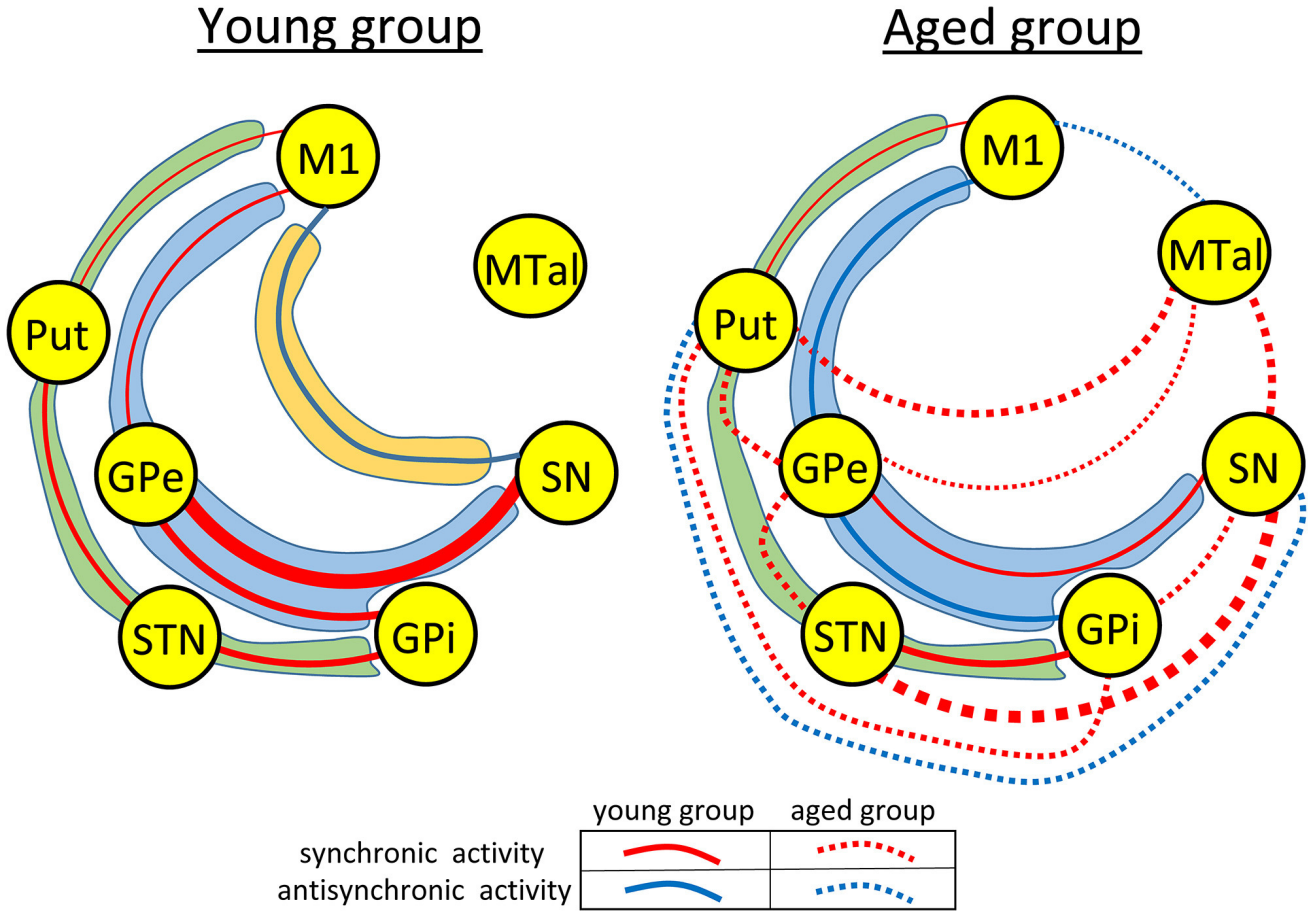
Diagrams showing a summary of the main functional networks of the basal ganglia motor circuit in the young- and aged-groups during the no-motion intervals. Functional relationships with statistical significance are shown with red (synchronic activity) or blue (anti-synchronic activity) lines. The amplitude of correlations is represented by the thickness of the lines connecting the different basal ganglia. In the aged-group (right-side hand), correlations found in both the aged and young groups are shown with continuous lines, and those found in the aged-group but not in the young-group are shown with discontinuous lines. The main functional networks found during the no-motion intervals are grouped within areas of different colors, the M1 ↔ Put ↔ STN ↔ GPi network in green areas, the M1 ↔ GPe ↔ GPi/SN network in blue areas, and the M1-SN network in an orange area. M1, primary motor cortex; Put, post-commissural putamen; GPe, external globus pallidum; STN, subthalamic nucleus; GPi, internal globus pallidum; SN, substantia nigral; and MTal, motor thalamus.

Thus, aging induced a marked reconfiguration of BG activity during the no-motion intervals which in many cases increased the synchronicity of BG (positive correlations), but which in some cases increased their anti-synchronic activity (negative correlations) or replaced their synchronic behavior by anti-synchronic behavior. A summary of these changes is shown in Figure 2, where the significant synchronicity of two nuclei is shown with red lines, and their anti-synchronicity with blue lines. Three chains of BG connections were found in the young-group during the no-motion intervals (Figure 2 left), a M1 ↔ Put ↔ STN ↔ GPi synchronic connection (green area), a M1 ↔ GPe ↔ GPi/SN synchronic connection (blue area), and an M1 ↔ SN anti-synchronic connection (orange area). In the aged-group (Figure 2 right): (1) M1 ↔ Put ↔ STN ↔ GPi synchronicity was replaced by M1 ↔ Put ↔ GPe ↔ STN ↔ GPi synchronicity, (2) M1 ↔ GPe ↔ GPi/SN synchronicity showed a marked decrease of GPe ↔ SN synchronicity and M1 ↔ GPe and GPe ↔ GPi synchronicity were replaced by anti-synchronic activities, and (3) M1-SN anti-synchronicity vanished. These changes were accompanied by a synchronic co-activation of Put-GPi, Put-MTal, GPe-MTal, GPi-SN, SN-MTal, and STN-SN and by an anti-synchronic activation of the Put-SN and MTal-M1.

### Functional Connectivity of BG During the Motor-Task

**Figure 3:**
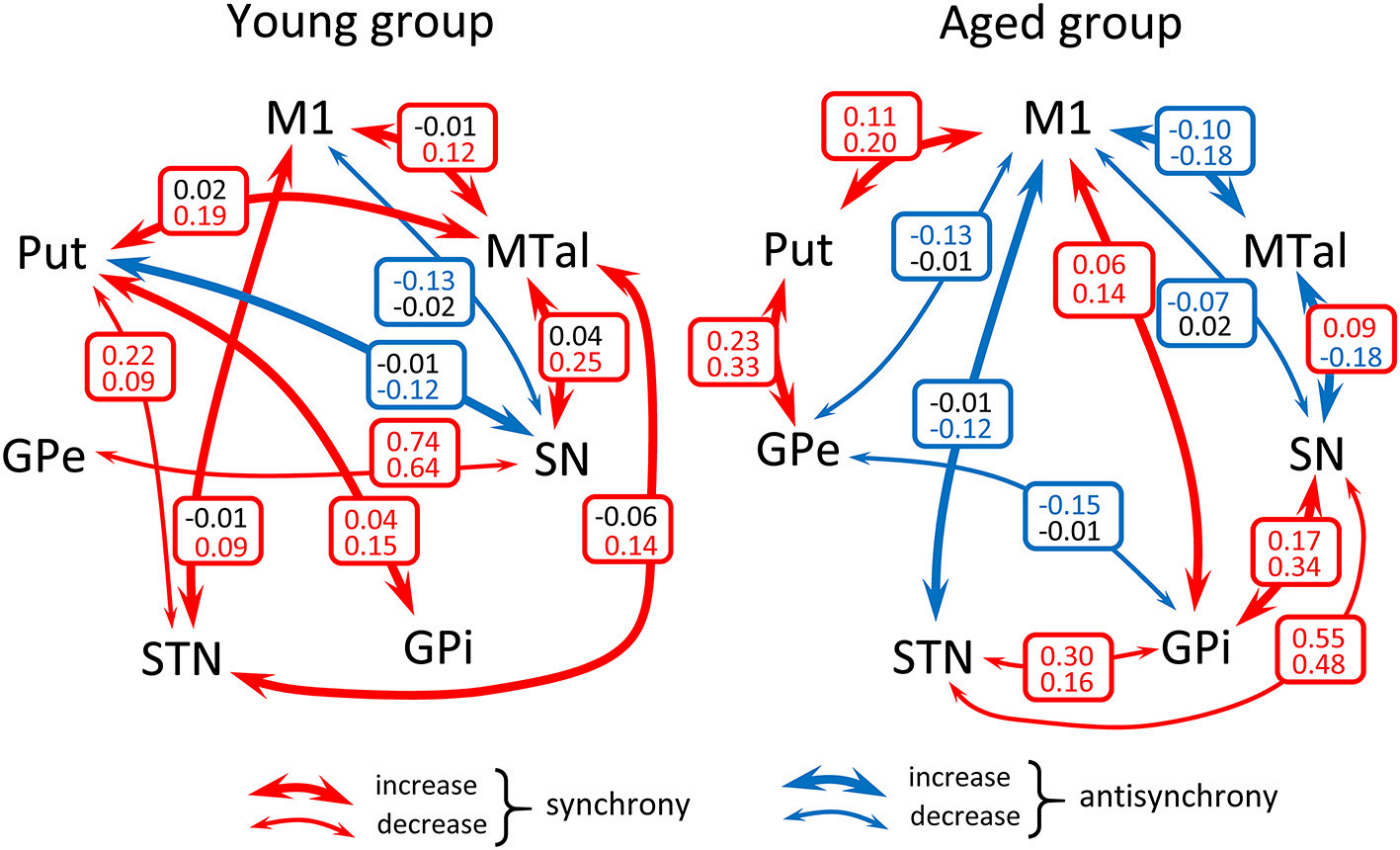
Diagrams showing a summary of the action of motor activity on the functional connectivity of BG in the young- and aged-groups. Numbers show the mean correlation coefficient for each group computed during the no-motion (top of squares) and the motion (bottom of squares) time-intervals in the young-group (left-side hand) and aged-group (right-side hand). Red numbers show significant positive correlations, blue numbers significant negative correlations and black numbers non-significant correlations. Significant differences of the correlation coefficient computed during task-resting and the motor-task are shown by asterisks. Only the functional relationships which changed with the motor activity are shown in this figure (all comparison reached a *p* < 0.001 statistical significance). Red arrows indicate significant changes of positive correlations and blue arrows significant changes of negative correlations. M1, primary motor cortex; Put, post-commissural putamen; GPe, external globus pallidum; STN, subthalamic nucleus; GPi, internal globus pallidum; SN, substantia nigral; and MTal, motor thalamus.

The influence of motion on the functional connectivity of BG was tested by comparing the CC computed during the motion and no-motion intervals. Figure 3 shows the CC values in the young-group (Figure 3 left) and aged-group (Figure 3 right), with the CC values computed for the no-motion and motion intervals shown at the top and bottom of each square, respectively. Red numbers show significant positive correlations, blue numbers significant negative correlations and black numbers non-significant correlations. Significant differences in CC computed between the no-motion and motion intervals (asterisk) indicate the effect of motion on the BG functional connectivity. Only the functional relationships which changed with the motor activity are shown in this figure. Red arrows indicate significant changes of positive CC and blue arrows significant changes of negative CC.

In the young-group, finger-movements increased the M1-STN, M1-MTal, Put-GPi, Put-MTal, STN-MTal, and SN-MTal synchronicity, increased the Put-SN anti-synchronicity, decreased the Put-STN and GPe-SN synchronicity, and decreased the M1-SN anti-synchronicity. The effect of motion was different in the aged-group, which showed an increase of M1-Put, M1-GPi, Put-GPe, and GPi-SN synchronicity, an increase of the M1-STN, M1-MTal, and SN-MTal anti-synchronicity, a decrease of STN-GPi and STN-SN synchronicity and a decrease of M1-GPe, M1-SN, and GPe-GPi anti-synchronicity.

## Discussion

The use of BOLD-data of VOIs located in the main nuclei of the BGmC and of the partial correlation method proved useful to study the effect of aging on the functional interaction of BG. BG showed marked changes in aged people during both the no-motion and motion intervals, suggesting that a different functional connectivity of BGmC nuclei may be at the basis of the motor deterioration induced by aging.

### Functional Connectivity of the BGmC in Young People

The classical BG-model is based on excitatory/inhibitory relationships between their nuclei (Alexander et al., 1986; Hoover and Strick, 1993; Delong and Wichmann, 2009). fcMRI does not provide information about the structural connectivity between centers or about the mechanisms involved in their functional interactions. Positive and negative BOLD-correlations do not indicate the existence of excitatory or inhibitory connections, and a high BOLD-correlation between two centers does not necessarily imply their “direct” interaction (the functional connectivity between two centers can be facilitated by other “crossing centers” able to transmit the information between each other). Despite these methodological constraints, fcMRI may reveal some aspects of the functional dynamic of BG that can go unnoticed for the tracing techniques that identify structural connections between brain centers and for the single-unit recordings that identify excitatory-inhibitory interactions between individual neurons of two brain centers. Motor tasks often need seconds to be executed, which allows the identification of *task-positive motor networks* with fcMRI methods (1.6 s is the time-resolution here). Previous studies have identified functional networks by detecting brain centers with synchronous BOLD-signal fluctuations and a positive correlation between the BOLD-signals of their centers (Biswal et al., 1995; Uddin et al., 2009; Tomasi and Volkow, 2010; Van Dijk et al., 2010; Tomasi et al., 2016). From this point of view, two centers showing no significant correlations do not belong to the same functional network (and could work in parallel without disturbing each other), and two centers showing a negative correlation (anti-synchronous BOLD-fluctuation) could belong to different networks with incompatible activities (Fox et al., 2005; Fair et al., 2007; Uddin et al., 2009; Hampson et al., 2010). In addition, motor networks may maintain their activity during the no-motion intervals, also working when subjects are not performing any voluntary motor task. Therefore, fcMRI is more suitable for analyzing the behavior of neuronal networks than for studying its “wiring” or the mechanisms involved in the interaction of their components. fcMRI data are more suitable to identify functional networks that to identify structural networks or to study the excitatory-inhibitory interactions between the centers of structural networks.

The young-group data suggest that the indirect pathway may work following two different functional arrangement, one involving a M1 ↔ Put ↔ STN ↔ GPi functional connectivity and the other involving a M1 ↔ GPe ↔ SN/GPi functional connectivity. M1 showed an anti-synchronous relationship with SN which suggests that the SN activation is followed by an M1 inactivation, a possibility which agrees with both the classical BG model (the GABAergic projections of SN to MTal inhibits the glutamatergic excitatory action of MTal on M1) (Alexander et al., 1986; Delong, 1990; Parent and Hazrati, 1995; Delong and Wichmann, 2009; Yin, 2017), and the fact that the inhibition of the SN activity (e.g., by inhibiting or lesioning the STN), and the fact that the inhibition of the SN activity (e.g., by inhibiting the STN) increases the M1 activity in Parkinson's disease (Obeso et al., 2000, 2017; Delong and Wichmann, 2009; Rodriguez-Rojas et al., 2018). The *functional networks* observed here during the no-motion intervals may be involved in the modulation of muscle tone, in the stabilization of body posture or in any function performed by BG when subjects are not executing voluntary motor patterns. The functional interaction of BG changed with the execution of the motor task. The synchronicity between most BG increased with voluntary movements (left-side Figure 3), showing that some nuclei of the BGmC are also involved in *task-positive motor networks*.

### Functional Connectivity of the BGmC in Aged People

The aging processes induced a substantial restructuration of the BG activity during the no-motion intervals. In the M1 ↔ Put ↔ STN ↔ GPi network, the Put ↔ STN synchronicity was replaced by the Put ↔ GPe ↔ STN synchronicity. In the M1 ↔ GPe ↔ SN/GPi network, M1 ↔ GPe ↔ GPi synchronicity was replaced by anti-synchronicity, and GPe ↔ SN synchronicity decreased. The SN-M1 anti-synchronicity observed in the young-group vanished in the aged-group. Additional interactions were observed in the aged-group, including a synchronicity between Put-MTal, Put-GPi, STN-SN, GPe-MTal, GPi-SN and SN-MTal, and anti-synchronicity between Put-SN and MTal-M1 (right-side Figure 3). The BGmC is involved in the stabilization of body posture (Takakusaki, 2017) and the modulation of muscle tone (Takakusaki et al., 2004), physiological functions that could be performed when subjects are not performing voluntary motor tasks. In this case, these motor problems of aged people (Woodhull-Mcneal, 1992; Agyapong-Badu et al., 2016) could be caused by a deficient functional connectivity of the *no-motion networks* of the BGmC.

The effect of motion on the BGmC was also different in the young and aged groups. BG synchronicities activated by motion in the young groups vanished in the aged group, with a number of anti-synchronicities and some new synchronicities not observed in the young group emerging in the aged-group (Figure 3). The BGmC is involved in the execution of unsupervised automatic motor patterns (Lehericy et al., 2005) which also decline with aging (Hellmers et al., 2018). The deficient functional connectivity of the *task-positive motor networks* of the BGmC observed here in the age-group could be at the basis of this behavioral problem.

The aged-group had an average age of 63 years old which was enough to find age-related differences with the young-group. These differences could be greater for older people but some misleading variables (e.g., instability of the motor task during the MRI study) could hamper the analysis of results, and therefore no person with more than 70 years of age was included in the study. The young and aged groups had similar compositions of men and women (8 men and 7 women in the young-group and 7 men and 8 women in the aged-group), and differences observed between the young and aged groups cannot be attributed to effects associated with gender. This does not mean that the effect of aging on BGmC is the same in both sexes, a possibility that would require a specific study. Different aged-related neurodegenerative diseases present muscle tone and motor behavior disorders. This is the case of Parkinson's disease, which normally shows slowness movements and muscle rigidity. It has been suggested that Parkinson's disease is produced by an accelerated aging of the brain, and particularly of the dopaminergic cells which control the basal ganglia motor circuit studied here (Rodriguez et al., 2014, 2015). Thus, it is possible that an increase of the aging effects on the basal ganglia motor circuit found here may be at the basis of some of the motor disorders of Parkinson disease.

In summary, present data provide evidence that the partial correlation of fcMRI data may be used to study the interactions of human BG when subjects are not performing voluntary movements, and to identify the modification of these interactions during the execution of particular tasks. This experimental approach even proved to be suitable to study complex closed-loop networks which, as occurs with the BGmC, present multiple structural and functional interactions between their components. The BGmC showed a generalized change of the functional connectivity of its center with aging, an effect observed during both the motion and the no-motion time-intervals. These changes may be at the basis of the movement and posture deterioration observed in age-related neurodegenerative disorders such as Parkinson's disease. New studies in aged people with particular motor handicaps are necessary to understand which BG interaction is involved in each of these problems. A similar study could be performed in Parkinson's patients with slowness movements (bradykinesia) or with a deterioration of the automatic activities which are necessary to maintain body posture.

## Data Availability Statement

The original contributions presented in the study are included in the article/supplementary material, further inquiries can be directed to the corresponding author/s.

## Ethics Statement

The studies involving human participants were reviewed and approved by an Institutional Review Board (Human Studies Committee-La Laguna University). The patients/participants provided their written informed consent to participate in this study.

## Author Contributions

CR-S was involved in the planning and execution of the study, in the recording of data, and in the manuscript review. IM was involved in the execution of the study. MR was involved in the planning of the study, analysis and interpretation of data, and in the manuscript review. All authors contributed to the article and approved the submitted version.

## Funding

This work was supported by the Center for Networked Biomedical Research in Neurodegenerative Diseases (CIBERNED; 2021/02), Madrid, Spain, and the Foundation Curemos el Parkinson, La Coruña, Spain.

## Conflict of Interest

The authors declare that the research was conducted in the absence of any commercial or financial relationships that could be construed as a potential conflict of interest.

## Publisher's Note

All claims expressed in this article are solely those of the authors and do not necessarily represent those of their affiliated organizations, or those of the publisher, the editors and the reviewers. Any product that may be evaluated in this article, or claim that may be made by its manufacturer, is not guaranteed or endorsed by the publisher.
